# CD133+ liver cancer stem cells resist interferon-gamma-induced autophagy

**DOI:** 10.1186/s12885-016-2050-6

**Published:** 2016-01-13

**Authors:** Jian Li, Jin-Na Chen, Ting-Ting Zeng, Fan He, Shu-Peng Chen, Stephanie Ma, Jiong Bi, Xiao-Feng Zhu, Xin-Yuan Guan

**Affiliations:** Department of Clinical Laboratory, Sun Yat-Sen Memorial Hospital, Sun Yat-Sen University, Room 102, 107 W. Yanjiang Road, Guangzhou, 510120 China; Guangdong Provincial Key Laboratory of Malignant Tumor Epigenetics and Gene Regulation, Sun Yat-Sen Memorial Hospital, Sun Yat-Sen University, Guangzhou, China; State Key Laboratory of Oncology in Southern China, Sun Yat-sen University Cancer Center, 651 Dongfeng Road East, Guangzhou, Guangdong 510060 PR China; Department of Clinical Oncology, Li Ka Shing Faculty of Medicine, The University of Hong Kong, Hong Kong, China; Department of Forensic Medicine, Zhongshan School of Medicine, Sun Yat-sen University, Guangzhou, China; Department of Surgery, The First Affiliated Hospital of Sun Yat-sen University, Guangzhou, China

**Keywords:** HCC, CSC, CD133, Immune, IFN-γ, Autophagy

## Abstract

**Background:**

Hepatocellular carcinoma (HCC) is one of the most fatal malignancies worldwide, and CD133 is a popular cancer stem cell (CSC) marker for HCC. CD133^+^ CSCs have been reported to resist conventional chemo- and radiotherapy, but little is known about their response to immune surveillance. Interferon-gamma (IFN-γ) is one of key cytokines that the immune system produce to eradicate cancer cells, so we investigated the function of IFN-γ on CD133+ HCC CSCs in this study.

**Methods:**

The response of CD133^+^ cells to IFN-γ was performed with functional assays (cell proliferation assay and tumor formation in nude mice), flow cytometry, immunofluorescence staining and RNA interference.

**Results:**

We found that IFN-γ inhibited the proliferation of cell lines with low percentage of CD133^+^ cells (wild-type human cells, BEL7402, QGY7701) but it did not affect the proliferation of cell lines with high percentage of CD133^+^ cells (wild-type human cells, Huh7, PLC8024) in vivo and in vitro (nude mice). Flow cytometry analysis demonstrated that the percentage of CD133+ cells increased after IFN-γ treatment of low CD133^+^ cell lines. Furthermore, IFN-γ induced the autophagy of low CD133^+^ cell lines to decrease proliferation.

**Conclusion:**

CD133^+^ HCC CSCs resisted IFN-γ-induced autophagy, which might also be a mechanism through which CSCs resist immune eradication.

**Electronic supplementary material:**

The online version of this article (doi:10.1186/s12885-016-2050-6) contains supplementary material, which is available to authorized users.

## Background

Because it is incurable, cancer is one of the most fatal diseases, and it affects millions of people worldwide [1]. Although significant efforts have been made by thousands of clinicians and researchers, no cure for cancer has been discovered [2–5]. Many cancers are resistance to radiotherapy and chemotherapy or have high recurrence after surgery or conventional treatment [6–10]. According to modern theory, cancer cells resist conventional therapy and cause recurrence due to the existence of a population of cancer cells termed cancer stem cells (CSCs) [11–17]. CSCs are considered the originating cancer cells, and they comprise a very small population of total cancer cells. However, this small population of cancer cells is also thought to be the origin of cancer recurrence and the primary cells that are resistant to radiotherapy and chemotherapy.

Hepatocellular carcinoma (HCC) first develops in the liver, and it is one of the ten most common cancers worldwide [18]. According to research from our group and others, CD133 is a highly recognized marker for HCC CSCs [19, 20]. CD133^+^ cells have strong in vivo tumorigenicity [19]. For example, 5 × 10^4^ CD133^+^ cells sorted from Huh7 cells could induce tumor formation in 7/9 of nude mice, while CD133^-^ cells could not induce tumor formation until the injecting cells up to 3 × 10^6^ cells. CD133^+^ HCC CSCs were reported to be resistant to doxorubicin and 5-fluoruracil-mediated apoptosis because of their preferential expression of the survival proteins involved in the Akt/PKB and Bcl-2 pathway [21]. CD133^+^ liver CSCs have also been reported to be resistant to radiotherapy because of their high activation of the MAPK/PI3K signaling pathway [22]. There have been few reports about the resistance of liver CSCs to immune eradication. However, some studies have suggested that such interactions might exist between CSCs and the immune system. For example, in an HCC nude mouse model, we observed several tumor cells that were surrounded by many immune cells in the local tumor region. This observation raised several questions, including the following: What are these cells’ characteristics? Are they liver CSCs? Because CD133 is an accepted surface marker for liver CSC [19, 20], we stained these tumor cells with a CD133 antibody and found that CD133^+^ tumor cells existed in this region.

The immune system plays a very important role in cancer immune surveillance [23]. Interferon-gamma (IFN-γ) is one of the most important cytokines that natural killer cells secrete to perform their defense duty in tumor immunity and against microbe invasion [24–27]. IFN-γ is a glycosylated protein that facilitates tumor rejection by modulating the systemic immunity to cancer cells. Thus, IFN-γ is a central modulator of tumor immunity, and it can also directly kill tumor cells by inducing cell apoptosis or autophagy after binding to receptors expressed on the tumor cell surface [28–34]. Although nude mice lack a thymus, large quantities of IFN-γ are still secreted by NK cells or other remaining immune cells. Thus, we investigated the direct function of IFN-γ on CD133^+^ liver CSCs in this study.

## Methods

### HCC cell lines and cell culture

The human HCC cell line Huh7 was provided by Dr. H. Nakabayashi, Hokkaido University School of Medicine, Japan. HCC cell lines PLC8024, BEL7402 and QGY7701 were obtained from the Institute of Virology of the Chinese Academy of Medical Sciences (Beijing, China). All HCC cell lines were cultured in high-glucose DMEM supplemented with 10 % FBS, 50 U/mL penicillin G and 50 μg/mL streptomycin under humidified conditions containing 5 % CO_2_ at 37 °C. No ethics approval was required for the use of above mentioned human HCC cell lines as it did not related to any ethics problem in the usage and passage of them in this study.

### Immunohistochemistry (IHC) staining

Paraffin-embedded, formalin-fixed nude mouse tumor tissue sections (5-μm-thick) were deparaffinized and rehydrated. Endogenous peroxidase activity was blocked with 3 % hydrogen peroxide for 30 min. For antigen retrieval, strict serial slides were immersed in 10 mM citrate buffer (pH 6.0) and boiled for 15 min in a microwave. Non-specific binding was blocked by 5 % bovine serum albumin (BSA) in phosphate buffer solution (PBS) for 30 min. Slides were then incubated with an antibody against human CD133 (1:100; Santa Cruz Biotechnology, Santa Cruz, CA, USA) or isotype at 4 °C overnight in a moist chamber. An HRP-conjugated secondary antibody was used to detect primary antibodies. Diaminobenzidine tetrahydrochloride was used as the visualization substrate, followed by counter staining with hematoxylin. Positively stained cells were observed and captured under a microscope.

### Hematoxylin and eosin (H.E.) staining

Paraffin-embedded, formalin-fixed tissue sections were deparaffinized and rehydrated. Nuclei were stained with hematoxylin, and the cytoplasm was developed with eosin after thorough washing.

### Cell proliferation assay and foci formation

Cell growth rates were determined using a Cell Counting Kit-8 (DOjinDO Laboratories, Japan) (CCK-8). Cells were seeded in a 96-well plate at a density of 5 × 10^3^ cells and incubated at 37 °C in a humidified atmosphere containing 5 % CO_2_. After 24 h, cultured cells were treated with different dosages of recombinant human IFN-γ (rhIFN-γ) (Peprotech, Rocky Hill, NJ, USA). The CCK-8 assay was then performed according to the manufacturer’s instructions. Three independent experiments were performed, and data were expressed as the mean ± SD. For the focus formation assay, 1 × 10^3^ cells were seeded in a 6-well plate and stimulated with or without 10 ng/ml(10000 IU/ml) rhIFN-γ for 2 weeks. Media were changed every three days. Surviving colonies were fixed and stained with 1 % crystal violet. Three independent experiments were performed.

### Effects of IFN-γ on in vivo proliferation

The study protocol was approved by and performed in accordance to the Committee of the Use of Live Animals in Teaching and Research at Sun Yat-sen University Cancer Center. 2×10^6^ PLC8024 and 5×10^6^BEL7402 cells were subcutaneously injected into nude mice. After two weeks, tumors were dissected and minced using a sterile blade. Minced tumors were then divided into ten parts and implanted into another ten nude mice. These ten nude mice were randomly divided into two groups. One group was IP injected with 20000 IU rhIFN-γ (CLONGAMMA, China) (to acquire the same IFN-γ concentration in vivo as that in in vitro culture) every day for four weeks, and the other group was injected with the same volume of buffer control. The tumor volume was measured weekly. After four weeks, mice were sacrificed and photographed, and tumors were minced and digested as described by Ma et al. [35] to acquire single cell suspensions for flow cytometry analysis.

### Flow cytometry analysis

For flow cytometry analysis, 1 × 10^6^ cells were acquired and washed once before staining with their respective antibody in 1 % BSA/PBS buffer for 30 min on ice in the dark. The following antibodies were used: PE-CD133/2 (Miltenyi Biotec; Auburn, CA, USA), FITC-IFN-γR1 (IFN-γ receptor 1) (R&D Systems, Minneapolis, MN, USA), APC-IFN-γR2 (IFN-γ receptor 2) (R&D Systems, Minneapolis, MN, USA), their respective isotopes from the same company were used as controls. Cells were analyzed on a flow cytometry (FC500, Beckman).

### Apoptosis analysis

1 × 10^6^ cells were stained in binding buffer, propidium iodide (PI) and FITC-conjugated Annexin V, as provided by the Annexin-VFLUOS Staining Kit (Roche Diagnostics), according to the manufacturer’s instructions. Cells were then analyzed by flow cytometry (FC500, Beckman).

### Immunofluorescence (IF) staining and confocal microscopy

Cells were seeded on glass cover slips in a 6-well plate at a density of 2 × 10^4^ cells and incubated at 37 °C in a humidified atmosphere containing 5 % CO_2_. After 24 h, cultured cells were treated with or without 10 ng/mL rhIFN-γ for 4 days. Media were changed every three days. Cells were washed twice with PBS and fixed in 4 % paraformaldehyde for 15 min at RT, and non-specific binding was blocked by 5 % BSA in PBS for 30 min at RT. Slides were incubated with a mouse antibody against human LC3 (1:100; MBL, Japan) at 4 °C overnight in a moist chamber. Unbound primary antibody was removed by washing slides twice in PBS for 5 min each. Alexa Fluor 488-conjugated anti-mouse immunoglobulin secondary antibodies were used for detection. Finally, cells were washed and mounted with mounting medium containing DAPI (Vector Laboratories, Burlingame, CA, USA) before being visualized by a confocal microscopy.

### RNA interference (RNAi) assay

BEL7402 and QGY7701 cells were transfected with small interference RNA (siRNA) against Atg5: Atg5 si-1: 5′-CAAUCCCAUCCAGAGUUGCUUGUGA-3′; Atg5 si-2: 5′-AGUGAACAUCUGAGCUACCCGGAUA-3′ (Shanghai GenePharma, China) or a scramble control using Lipofectamine 2000 reagent (Invitrogen, Carlsbad, CA, USA) according to the manufacturer’s instruction. Transfected cells were treated with 10 ng/mL rhIFN-γ for four days; the gene silencing efficiency and autophagy were measured by western blotting. Cell growth was detected using a CCK-8 assay.

### Quantitative real-time PCR (Q-PCR)

Total RNA was extracted using TRIzol Reagent (Invitrogen, Carlsbad, CA), and reverse transcription was performed using an Advantage® RT for PCR Kit (Clontech, Mountain View, CA) according the manufacturer’s instructions. For Q-PCR analysis, aliquot of double-stranded cDNA was amplified with the primer of CD133: (Fw: 5′-TGGATGCAGAACTTGACAACGT-3′, Rv: 5′-ATACCTGCTACGACAGTCGTGGT-3′), or 18S: (5′-CTCTTAGCTGAGTGTCCCGC-3′, Rv: 5′-CTGATCGTCTTCGAACCTCC-3′) using a SYBR Green PCR Kit (Applied Biosystems, Carlsbad, CA) and an ABI PRISM 7900 Sequence Detector. CD133 mRNA delta Ct = CD133 Ct-18S Ct. All reactions were performed in duplicate.

### Western blotting (WB) analyses

Whole cell lysates were harvested with cell lysis buffer according to the manufacturer’s instructions. Western blotting analyses were performed with the standard protocol using antibodies against human GAPDH (Santa Cruz Biotechnology, Santa Cruz, CA, USA), LC3 (Novus, Littleton, CO, USA) and P62 (Cell Signaling Technology, Beverly, MA, USA).

### Statistical analysis

Comparisons between groups were analyzed using Student’s *t*-test or One-Way ANOVA for more than two groups in the SPSS software (version 16.0). A value of *P* < 0.05 (two-tailed) was considered statistically significant.

## Results

### CD133^+^ cells were detected in mice eight weeks after HCC cell injection

**Fig. 1:**
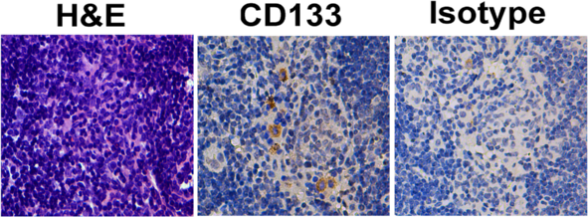
H.E. and IHC staining of PLC8024 cells implanted tumor spots. H.E. and IHC staining for CD133 and isotype expression in strict serial PLC8024 cells implanted tumor spot sections (400×)

To study whether cancer cells can live for long periods of time in nude mice, 1×10^4^ PLC8024 cells were subcutaneously injected into nude mice. Eight weeks after injection, we obtained local implanted tissues from treated mice. We observed cancer cells surrounded by many immune cells by H&E staining (Fig. 1). To confirm whether HCC CSCs existed in this spot or not, we stained tissues with the HCC CSC marker CD133. Results showed that a subset of tumor cells were CD133^+^ (Fig. 1), indicating that CSCs could live for long periods of time in tested animals.

### CD133^+^ HCC cells resisted IFN-γ-induced growth delay

**Fig. 2:**
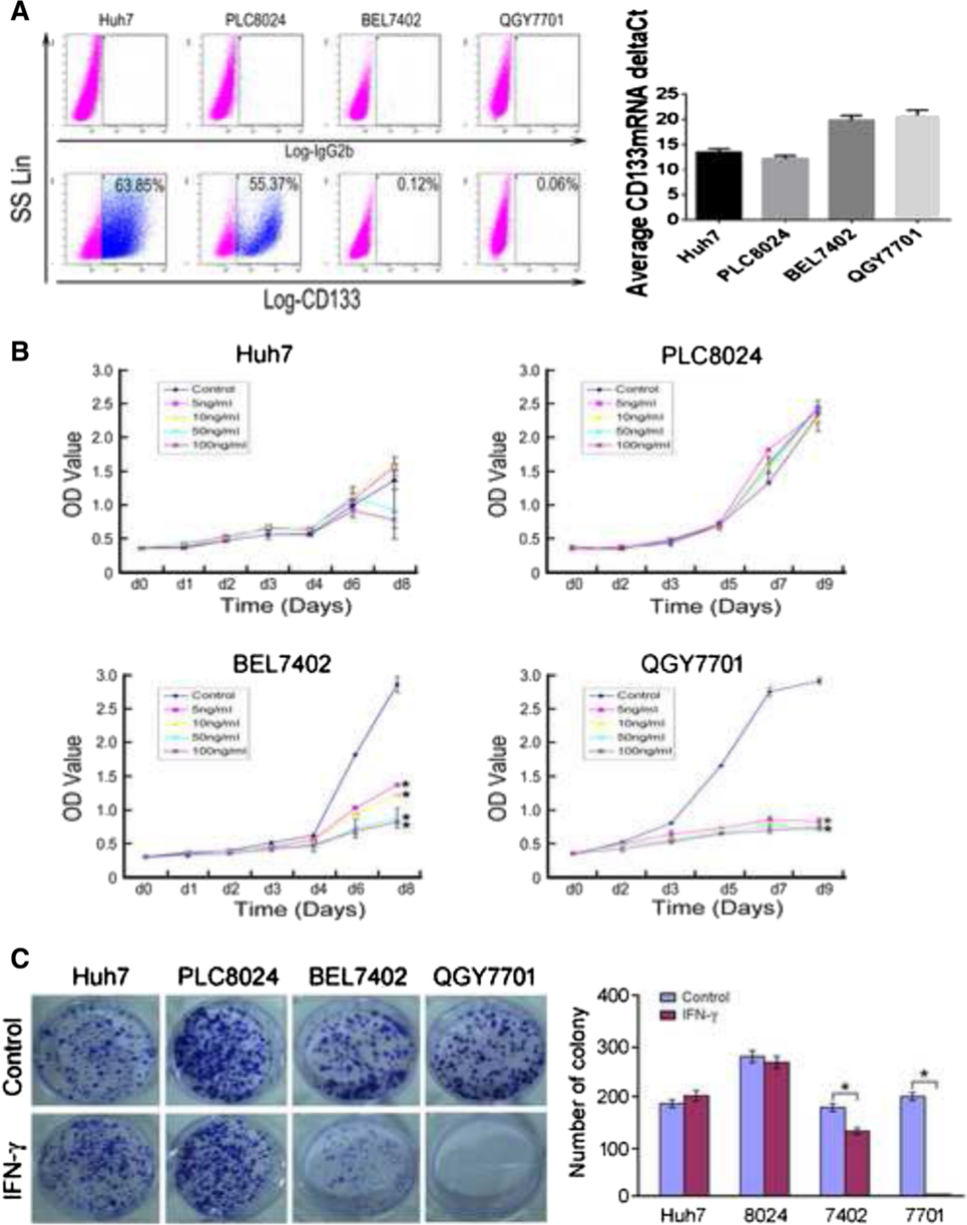
CD133 expression and proliferation assay of IFN-γ-treated HCC cell lines. **a** Left, flow results of CD133 expression in four different cell lines. Right, Q-PCR results of CD133 expression in four different cell lines. **b** CCK-8 assay of different IFN-γ dosages in various HCC cell lines. *, *p* < 0.05. **c** Foci formation assay of four HCC cell lines treated with or without 10 ng/ml IFN-**γ** for two weeks. Left, representative graph; Right, summary figure.*, *p* < 0.05. Representative of three independent experiments

**Fig. 3:**
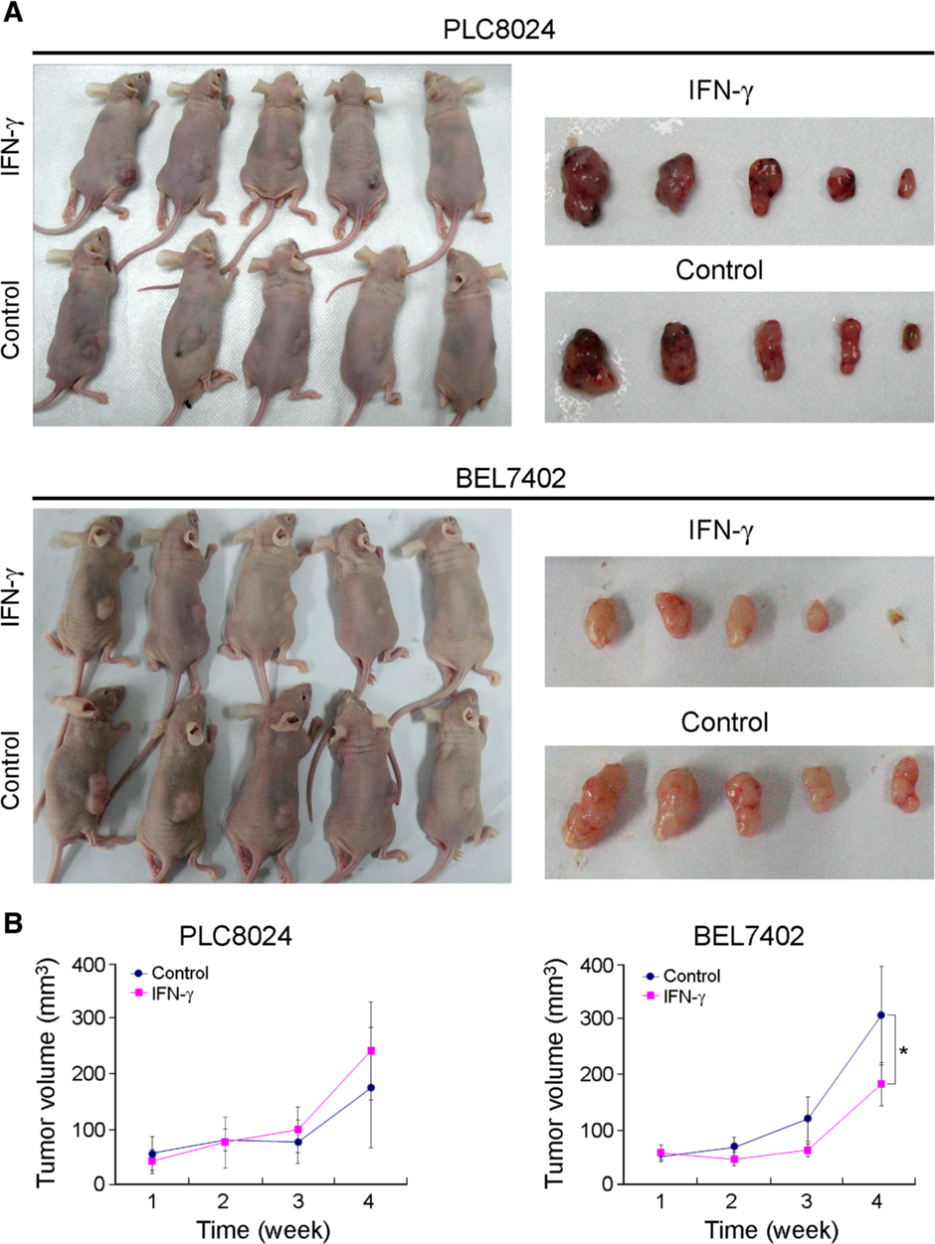
*In vivo* effect of IFN-γ on PLC8024 and BEL7402 cell-implanted nude mice. **a** Photograph of PLC8024 and BEL7402 implanted nude mice treated with or without IFN-γ for four weeks. **b** Tumor volumes in PLC8024 and BEL7402-implanted nude mice treated with or without IFN-γ, measured weekly. *, *p* < 0.05

As liver CSCs were surrounded by many immune cells, we continued to explore whether they resisted to attack induced by IFN-γ or not, which is an important cytokine secreted by immune cells. Four HCC cell lines with different percentages of CD133^+^ cells [19] (Fig. 2a) were treated with different dosages of rhIFN-γ. The cell growth rate was determined by a CCK-8 assay, and results showed that HCC cell lines with a high percentage of CD133^+^ cells (Huh7 and PLC8024) were more resistant to rhIFN-γ treatment than were HCC cell lines with a low percentage of CD133^+^ cells (BEL7402 and QGY7701) (Fig. 2b). Foci formation assays also demonstrated that HCC cell lines with a high percentage of CD133^+^ cells had a stronger resistance than did HCC cell lines with a low percentage of CD133^+^ cells (Fig. 2c). *In vivo* tumor formation assays also demonstrated that PLC8024 cells were more resistant to IFN-γ treatment compared with BEL7402 cells (Fig. 3).

### IFN-γ treatment enriched the CD133^+^ cell population in vitro and in vivo

**Fig. 4:**
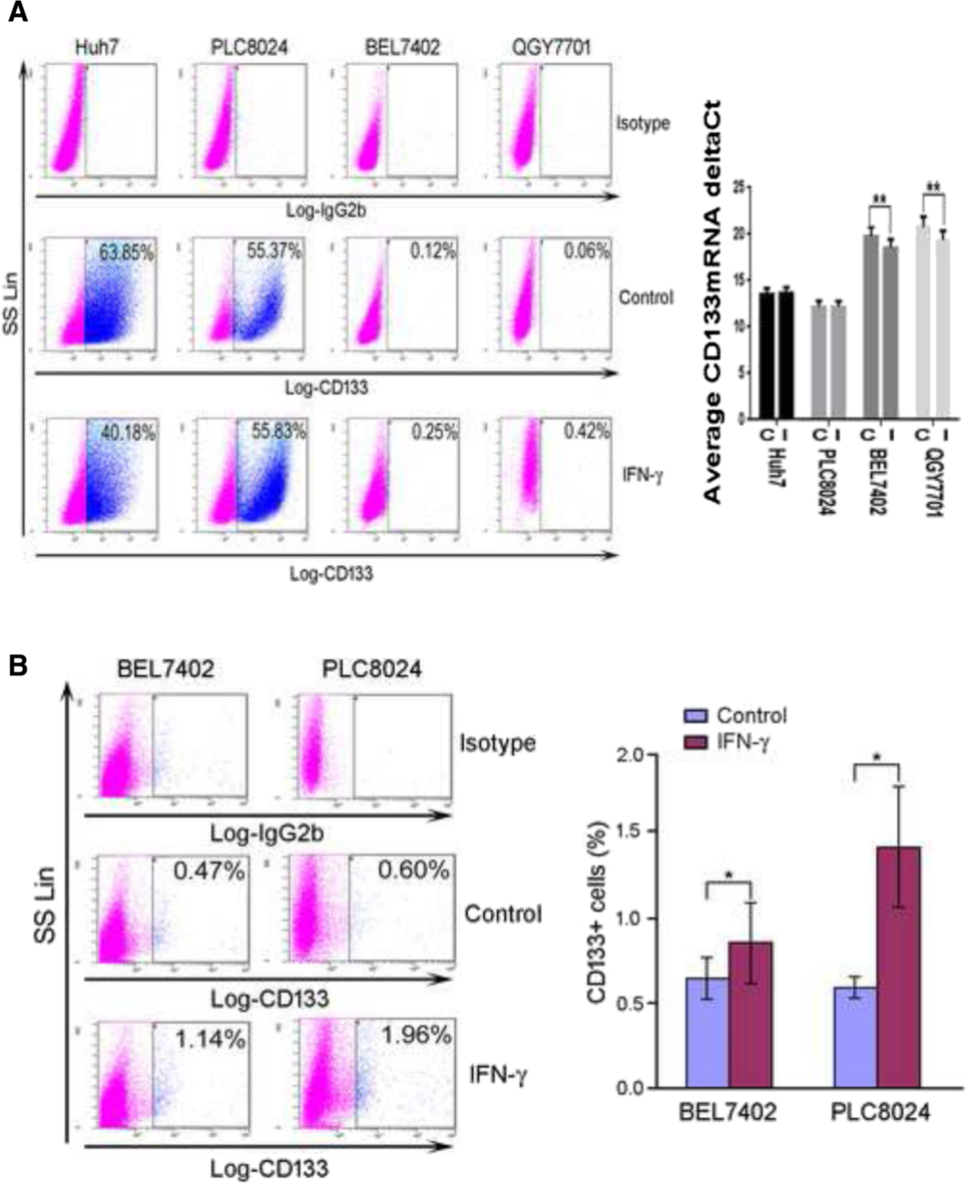
Expression of CD133^+^ cells after IFN-γ treatment *in vitro* and *in vivo*. **a** Left, representative percentage of CD133^+^ cells in four HCC cell lines with or without 10 ng/ml IFN-γ treatment for four days analyzed by flow cytometry. Control, treated with buffer control; IFN-γ, treated with 10 ng/ml IFN-γ. Right, Q-PCR results of CD133 mRNA expression in four HCC cell lines with or without 10 ng/ml IFN-γ treatment for four days. C, treated with buffer control; I, treated with 10 ng/ml IFN-γ. **, *p* < 0.01. Representative of three independent experiments. **b** Left, representative percentage of CD133^+^ cells in the indicated cell line-implanted nude mice with or without IFN-γ treatment for four weeks analyzed by flow cytometry. Right, summary figure.*, *p* < 0.05. Gating strategy, all gating of live tumor cells

To test whether IFN-γ treatment can enrich the CD133^+^ cell population or not, we determined the percentage of CD133^+^ cells in BEL7402, QGY7701, Huh7 and PLC8024 cell lines by flow cytometry and Q-PCR after IFN-γ (10 ng/ml) treatment. Results demonstrated that the percentage of CD133+ cells in BEL 7402 was doubled and the percentage of CD133+ in QGY 7701 was increased by seven times after IFN-γ treatment. IFN-γ had no significant effect on PLC8024 cells. In contrast, the percentage of CD133^+^ Huh7 cells slightly decreased after IFN-γ treatment (Fig. 4a). After we found that IFN-γ influenced differently on different HCC cell line *in vitro*, we wanted to explore what happened *in vivo*. So we followed to test whether IFN-γ treatment could increase the CD133+ cell population in xenograft tumors induced by PLC8024 or BEL7402 cells or not. For each cell line, 5 xenograft nude mice were treated with or without IFN-γ. Interestingly, the percentage of CD133^+^ cells significantly increased in xenograft tumors treated with IFN-γ compared with the control tumors without IFN-γ treatment (*P* < 0.05, Fig. 4b).

### CD133^+^ cells resisted IFN-γ induced apoptosis and autophagy

**Fig. 5:**
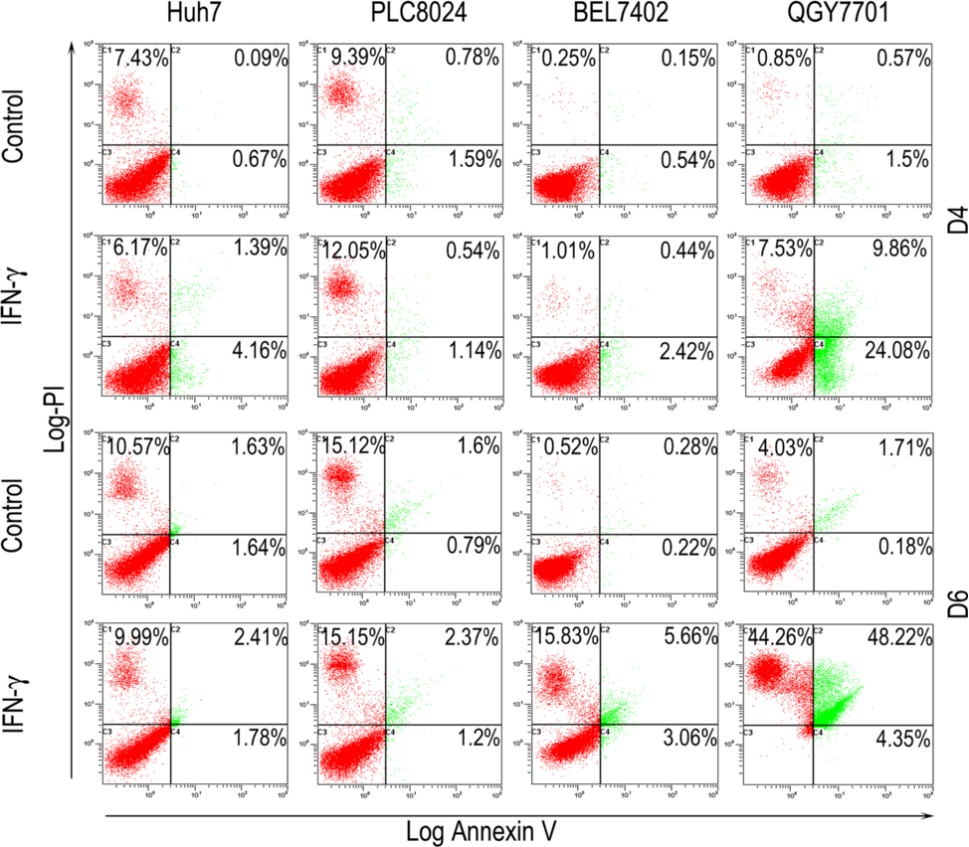
Apoptosis analysis in four cell lines after 10 ng/ml IFN-γ treatment for the indicated times analyzed by flow cytometry. Gating strategy, all gating of tumor cells. Representative of three independent experiments

**Fig. 6:**
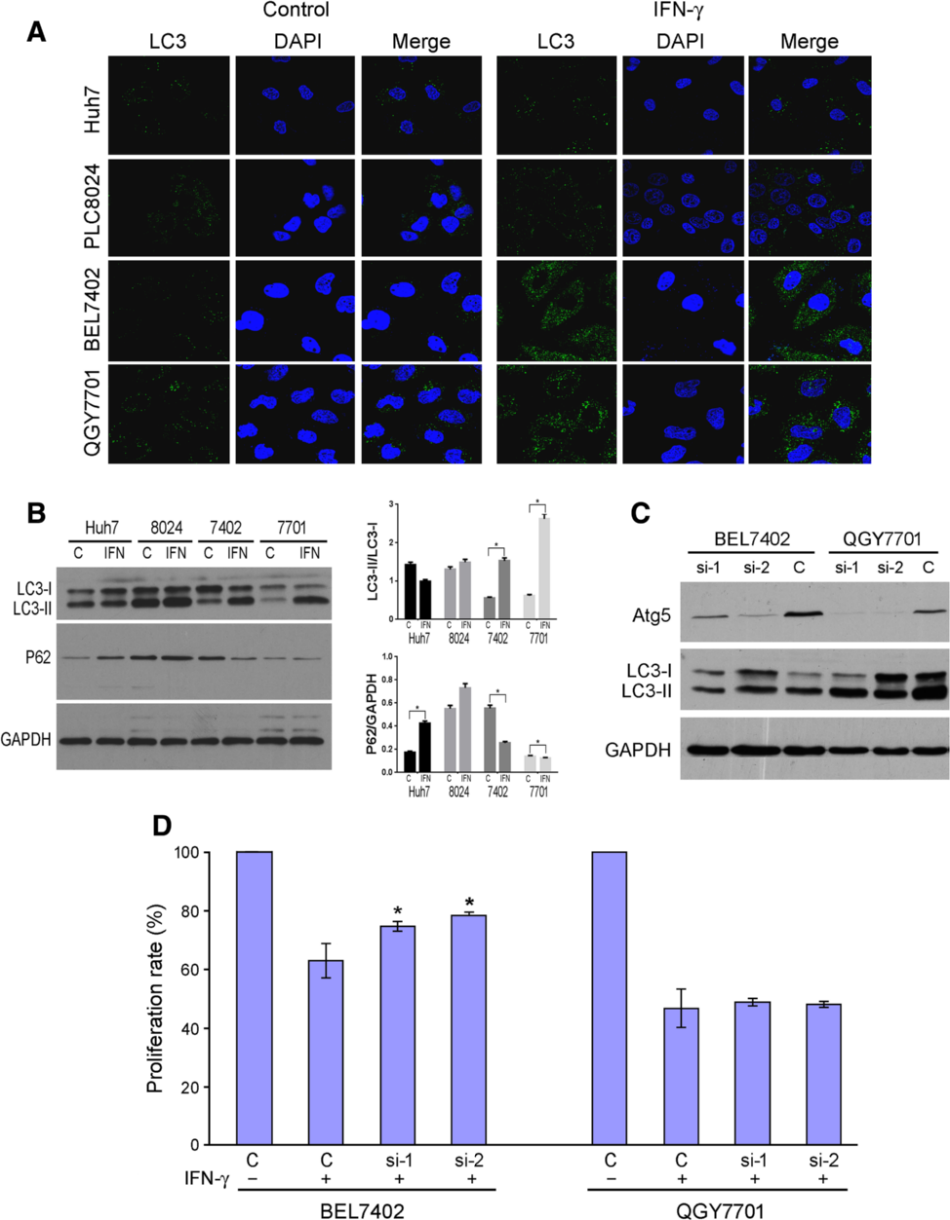
IFN-γ increased the autophagy of BEL7402 and QGY7701. **a** LC3 distribution in four cell lines treated with or without 10 ng/ml IFN-γ for four days detected by confocal. **b** Left, immunoblotting results of LC3 and p62 expression in four cell lines treated with or without 10 ng/ml IFN-γ for four days. Right, expression of indicated molecular were semi-quantitative analyzed with imageJ. 8024, PLC8024; 7402, BEL7402; 7701, QGY7701. C, control; IFN, IFN-γ. *, *p* < 0.05. **c** immunoblotting results of LC3 expression in BEL7402 and QGY7701 treated with or without 10 ng/ml IFN-γ for four days after Atg5 siRNA knockdown. **d** proliferation assay of BEL7402 and QGY7701 cells treated with 10 ng/ml IFN-γ after Atg5 knockdown detected by CCK-8 assay. si-1, siAtg5-1; si-2, siAtg5-2. *, *p* < 0.05. Representative of three independent experiments

Because IFN-γ can induce tumor cell apoptosis [34], we tested whether CSCs could resist IFN-γ-induced apoptosis in HCC cell lines or not. The apoptotic index was detected by flow cytometry at day 4 and 6 after IFN-γ treatment (10 ng/ml). Results showed that IFN-γ only induced apoptosis in QGY7701 cells (Fig. 5), suggesting that BEL7402, Huh7 and PLC8024 cells were resistant to IFN-γ-induced apoptosis. Because IFN-γ can also cause cell death by inducing autophagy [35], we next studied whether HCC CSCs could resist IFN-γ-induced autophagy or not. Results from both immunofluorescence staining (Fig. 6a) and Western blot (Fig. 6b) analysis demonstrated that IFN-γ treatment increased autophagy in BEL7402 and QGY7701 cells, but not in the Huh7 and PLC8024 cells.

### Atg5 knockdown prevented IFN-γ-induced autophagy in low CD133^+^ HCC cell lines

To further verify that IFN-γ induced growth delay was mediated by IFN-γ-induced autophagy, we knocked down Atg5 expression, which is an important component of the autophagy pathway [36, 37]. Results showed that Atg5 knockdown prevented IFN-γ induced autophagy in BEL7402 and QGY7701 (Fig. 6c). However, knocking down Atg5 reversed IFN-γ induced growth arrest in the BEL7402 cell line, but not in the QGY7701 cell line (Fig. 6d).

### IFN-γ receptors were not differently expressed between high and low CD133+ percentage HCC cell lines

Because different cell lines’ responses to IFN-γ treatment segregated with their *CD133*+ cell content, we examined whether their response could be explained by IFN-γ receptor expression or not. Thus, we detected IFN-γ receptor expression on the cell surface using flow cytometry. Results demonstrated that cells did not have different expression levels of IFN-γ receptors on their surfaces (Additional file 1: Figure S1).

## Discussion

CSCs have been reported to cause tumor recurrence, resist chemotherapeutic reagent and radiotherapy-induced apoptosis [21, 22]. In the current investigation, we demonstrated that CD133^+^ liver CSCs resisted IFN-γ-induced autophagy, which might be a novel defining characteristic of liver CSCs. Of course, tumor cells that were resistant to IFN-γ induced autophagy should not be limited to CD133^+^ CSCs because we also found that many CD133^-^ tumor cells remained after IFN-γ treatment both *in vivo* and *in vitro*. Thus, other mechanisms or characteristics of tumor cells might exist to resist IFN-γ-induced cell death.

In this investigation, we observed the phenomenon that high percentage CD133^+^ PLC8024 cell line *in vitro* changed to low percentage of CD133^+^ cell *in vivo*, which was in accordance with what had been reported about Huh7 [19]. The reason might be that a tumor hierarchy existed in cancer stem cells in which the CD133^+^ cells might generate CD133^-^ tumor cells [19]. The difference that we did not observed significant increase of CD133^+^ percentage *in vitro* in PLC8024 and observed the enrichment of CD133^+^ cells *in vivo* might be that the percentage of PLC8024 cell line *in vitro* was very high and it was hard to observe the significant increase, whereas the *in vivo* CD133^+^ percentage was very low and it was easy to observe the difference.

Ma et al. previously reported that either CD133^-^ or CD133^+^ cells separated by sorting maintained the normal CD133^+^ cell percentage level after short-term culture [19]. Furthermore, the significantly different cellular reactions to IFN-γ treatment were not apparent until four days in culture. Thus, we did not observe significantly different reactions to IFN-γ treatment between CD133^+^ and CD133-negative cells sorted from Huh7 or PLC8024 cell lines (data not shown).

IFN-γ is an important component of the innate and cellular immune systems for attacking tumors. There have been many reports about the function of IFN-γ on tumor cells. IFN-γ can induce the upregulation of tumor-associated antigens, such as carcinoembryonic antigen and TAG72, to enhance the immunogenicity of tumor cells [38]. It can also directly induce tumor cell apoptosis or autophagy [30, 33, 34]. In this investigation, we found that IFN-γ can induce autophagy in low CD133^+^ percentage cell lines, but not that in high CD133^+^ percentage cell lines. Furthermore, we detected an increase in the percentage of CD133^+^ cells in low CD133^+^ percentage cell lines after IFN-γ treatment, which suggested that CD133^+^ cells might resist IFN-γ induced autophagy. These results also implied that to completely eliminate cancer from the body, treatment with only IFN-γ is insufficient because a portion of CD133^+^ CSCs were resistant to IFN-γ. These data may partially explain why some patients demonstrated little or no response to IFN-γ treatment on clinic [39]. High expression of Bcl-2 was reported to be responsible for the apoptosis or autopahgy resistance induced by IFN-γ in human tumor-derived endothelial cells or human lung epithelial A549 cells [40, 41]. And Bcl-2 was also reported to be high expressed in CD133^+^ CSCs [21], which might be the potential mechanism of CD133^+^ CSCs resisted to IFN-γ induced apoptosis and autophagy in this study.

In this investigation, we also found that IFN-γ could induce both apoptosis and autophagy in QGY7701 cell line. Whereas it could only induce autophagy in BEL7402 cell line. So IFN-γ induced cell growth delay in QGY7701 might be due to the apoptosis and autophagy induced by IFN-γ in QGY7701’s CD133^-^ cells and IFN-γ induced cell growth delay in BEL7402 might be due to the autophagy induced by IFN-γ in BEL7402’s CD133^-^ cells. Thus, when we knocked down the expression of Atg5 in BEL7402, IFN-γ induced autophagy was inhibited. So IFN-γ induced cell growth delay was restored. Whereas in QGY7701 cell line, even we blocked IFN-γ induced autophagy by knocking down the expression of Atg5, IFN-γ could still delay their growth by inducing the apoptosis of QGY7701’s CD133^-^ cells. So knocking down the expression of Atg5 could not restore IFN-γ induced cell growth delay in QGY7701.

## Conclusion

We reported for the first time that CD133^+^ cancer stem cells existed in microenvironment surrounded by many immune cells in nude mice. Further investigated explored that CD133+ CSCs could resist IFN-γ induced autophagy *in vivo* and *in vitro*. These findings may add new characteristics to cancer stem cells and help to explore their key role in tumor surveillance evasion.

## Additional file

Additional file 1: Figure S1. Flow cytometry analysis of IFN-γ receptor surface expression in four cell lines. The dotted lines represented the isotype control. Representative of three independent experiments. (TIF 418 kb)

## Abbreviations

CCK-8: cell counting kit-8
CSC: cancer stem cell
H.E.: Hematoxylin and Eosin
HCC: hepatocellular carcinoma
IF: immunofluorescence
IFN-γR1: IFN-γ receptor1
IFN-γR2: IFN-γ receptor 2
IHC: immunohistochemistry
PI: propidium iodide
Q-PCR: quantitative real-time PCR
rhIFN-γ: human IFN-γ
RNAi: RNA interference
siRNA: small interference
WB: western blotting
