# Supplementary figures and images for: CD133+ liver cancer stem cells resist interferon-gamma-induced autophagy
Source: BMC Cancer. 2016 Jan 13;16:15. doi: 10.1186/s12885-016-2050-6 (PMC4711109; doi:10.1186/s12885-016-2050-6)

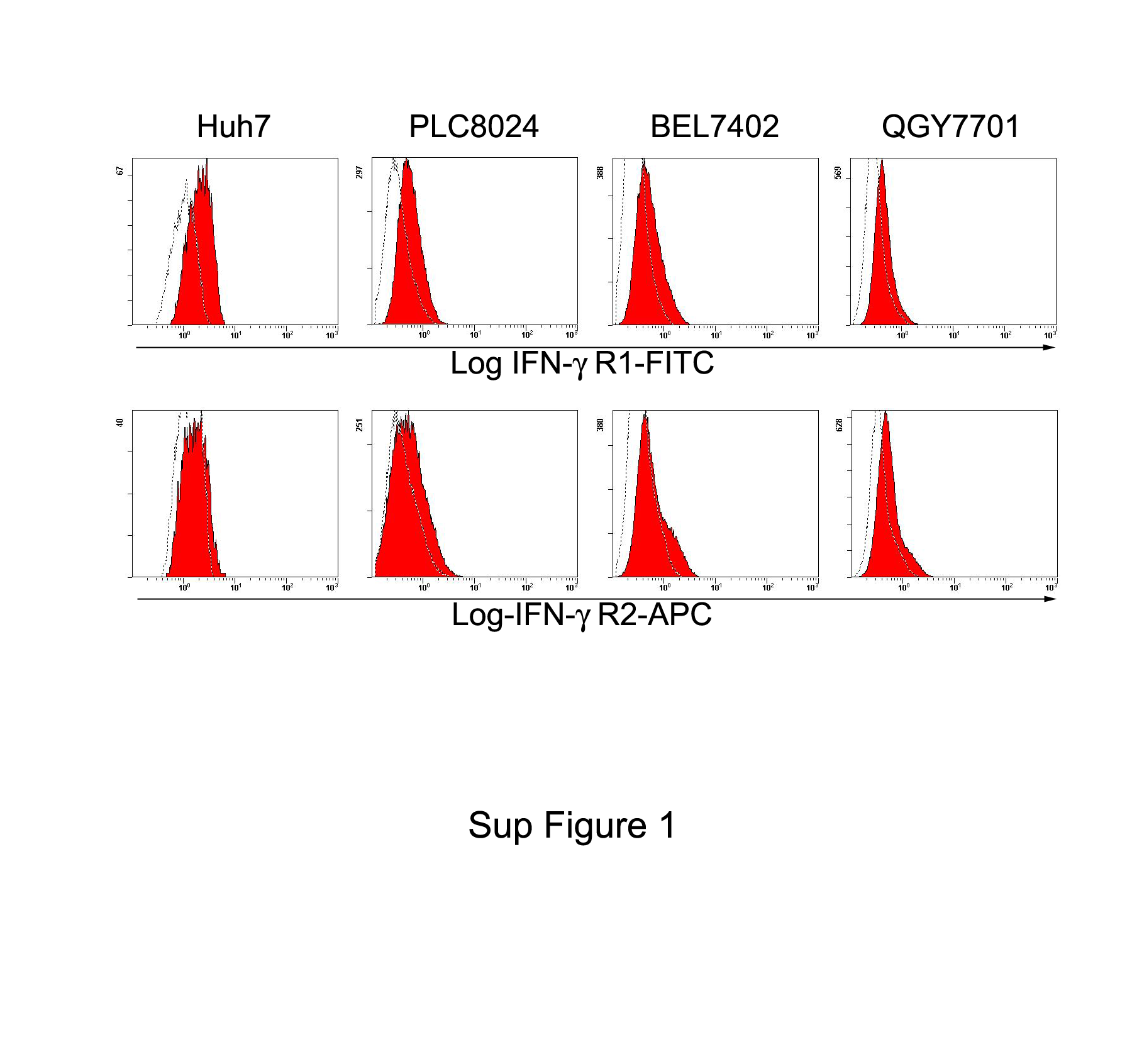

Supplement: Additional file 1: Figure S1. — Flow cytometry analysis of IFN-γ receptor surface expression in four cell lines. The dotted lines represented the isotype control. Representative of three independent experiments. (TIF 418 kb) [file 12885_2016_2050_MOESM1_ESM.tif]
